# Cryopreservation and re-culture of a 2.3 litre biomass for use in a bioartificial liver device

**DOI:** 10.1371/journal.pone.0183385

**Published:** 2017-08-25

**Authors:** Peter Kilbride, Stephen Lamb, Stephanie Gibbons, James Bundy, Eloy Erro, Clare Selden, Barry Fuller, John Morris

**Affiliations:** 1 Asymptote, General Electric Healthcare, Cambridge, United Kingdom; 2 Institute for Liver and Digestive Health, Royal Free Hospital Campus, University College London, London, United Kingdom; 3 Department of Surgery, Royal Free Hospital Campus, University College London, London, United Kingdom; Kyoto Daigaku, JAPAN

## Abstract

For large and complex tissue engineered constructs to be available on demand, long term storage using methods, such as cryopreservation, are essential. This study optimised parameters such as excess media concentration and warming rates and used the findings to enable the successful cryopreservation of 2.3 litres of alginate encapsulated liver cell spheroids. This volume of biomass is typical of those required for successful treatment of Acute Liver Failure using our Bioartificial Liver Device. Adding a buffer of medium above the biomass, as well as slow (0.6°C/min) warming rates was found to give the best results, so long as the warming through the equilibrium melting temperature was rapid. After 72 h post thaw-culture, viable cell number, glucose consumption, lactate production, and alpha-fetoprotein production had recovered to pre-freeze values in the 2.3 litre biomass (1.00 ± 0.05, 1.19 ± 0.10, 1.23 ± 0.18, 2.03 ± 0.04 per ml biomass of the pre-cryopreservation values respectively). It was also shown that further improvements in warming rates of the biomass could reduce recovery time to < 48 h. This is the first example of a biomass of this volume being successfully cryopreserved in a single cassette and re-cultured. It demonstrates that a bioartificial liver device can be cryopreserved, and has wider applications to scale-up large volume cryopreservation.

## Introduction

Acute Liver Failure (ALF) is a rare but extremely serious condition, where patient death can occur within weeks of diagnosis [1–3]. The only established treatment for ALF is organ transplant, which comes with the associated problems related with organ rejection and long term immunosuppressant-related illnesses. The survival outcome for patients with ALF receiving a transplant is lower than for patients receiving an elective liver transplant, and patients presenting with ALF are often not candidates for transplantation [1–3]. A bioartificial liver (BAL) may fill this unmet clinical need by providing extra-corporeal liver support allowing a patient’s own liver to recover from injury and removing the need for transplant [4, 5].

It takes up to 26 days of cell culture to grow sufficient biomass required for a BAL. This biomass cannot currently be stored for more than a few days without cryopreservation. However, ALF can prove fatal within days to weeks of symptoms first appearing, and so a cryopreservation protocol is essential to ensure on-demand access to BAL treatment [3, 6–11].

In our BAL cells are encapsulated into alginate to form encapsulated liver spheroids (ELS). This encapsulation increases cell number and functional performance of the cells (ELS) [12]. The alginate may also provide some benefit to cryopreservation outcome [13–15]. The total volume of ELS in our BAL device is just over 2 litres, which contains approximately 30% of the cell number of an adult human liver, and is projected to be sufficient to provide full liver support [5]. Cryopreservation of this large sample volume exhibits many differences compared with cryopreservation at smaller volumes [6].

The focus in cryopreservation of cells until now has largely been on small volumes, typically up to 1 ml samples. Exceptions are the cryopreservation of adult stem cells and T-cell therapies, typically in volumes 50-500ml, and ELS and other cell types in cryobags. There have also been reports of successful cryopreservation of sheep ovaries (1cm^3^ approximately [16, 17]), and the vitrification of a rabbit kidney (approximately 10cm^3^ [18]) [17, 19, 20]. We have found no record of biomasses larger than 500ml cryobags (for cell suspensions), or volumes larger than a few hundred ml for biomasses more complex than a cell suspension being cryopreserved in the frozen state, and these tend to be flattened for cryopreservation in morphologies that would be impractical for larger volumes or rigid constructs [8, 17, 21–23]. This situation exists despite the growing unmet demand for the preservation of tissue engineered constructs that cannot be produced using Just-In-Time manufacture, neither economically nor logistically [7, 19].

The study of large volume cryopreservation is also essential to improve transplant results. Currently organs can only be preserved in a chilled state (typically between 0–4°C) for a maximum of between 4–24 h, depending on the organ [24–26]. This greatly inhibits successful transplant numbers and outcomes. For example, currently almost half of donor hearts are discarded, with only 1 in 3 being used for transplant [27]. Increasing the transportation window through organ banking should increase transplantation rate. Work towards large volume cryopreservation in cell spheroids is an important step towards large scale organ banking, which combined with tissue engineering could ultimately prevent 30–35% of deaths annually in the United States alone [7].

### Different ice structures

Previously, work has examined the differences in ice structure between large and small volumes [6]. Small volumes (up to a few ml), tend to undercool below their equilibrium melting point [28]. Ice then nucleates and spreads rapidly throughout the sample, leading to a dendritic ice structure termed network solidification (NS).

Alternatively, in a larger volume, ice tends to nucleate at a higher temperature. As ice starts to form, the latent heat of solidification is released, the sample temperature will rise to the equilibrium melting point, and ice will then form more slowly, demonstrating a larger more structured ice form, in a process known as progressive solidification (PS) [6, 29].

PS can be introduced in smaller volumes such as a cryovial by using slow rates of cooling preferably at one wall of the vessel and adding an ice nucleant, that acts as a catalyst for ice formation. Thus the relative amount of supercooling is reduced to a small volume adjacent to the wall [5, 24, 28].

### Experimental design

Initially, two studies were carried out exploring the effects of a buffer medium and warming protocols to optimise ELS cryopreservation.

Three further cryopreservation conditions were then carried out.

The primary aim of this study was the condition allowing cryopreservation and post-thaw culture of a 2.3 litre biomass consisting of ELS.

In order to separate the study of cooling and warming effects during cryopreservation the two remaining conditions were carried out. These examined the post-thaw outcome of small volume (2ml) samples cryopreserved in cryovials (experiencing network solidification on cooling), and the post-thaw outcome for samples experiencing the same conditions as in the cooling phase in large volume cryopreservation, but being warmed more rapidly than is possible with a large volume using the scale down process system.

This study uses the abbreviations LV (large volume, 2.3 litre biomass), CV (2ml cryovial cryopreservation), and SDP (scale down process cryopreservation) to refer to each of these conditions.

## Methods

### Cell encapsulation and culture

HepG2 cells were obtained from the ECACC (Wiltshire, UK), and were grown in monolayer, before being passaged and encapsulated into 1% alginate beads, diameter 500μm, using a GeniaLab Jetcutter device, with final density 2 million cells/ml and 0.75% glass beads (w/v Kisker Biotech, Steinfurt, Germany) added for buoyancy. The 2.3 litres of biomass were cultured in a fluidised bed bioreactor (FBB) for 12 days, with partial media changes every 2–3 days.

ELS were cultured in alpha-MEM medium, supplemented with 50 U/ml penicillin, 50 ug/ml streptomycin (Invitrogen plc. Carlesbad, CA, USA), and 10% v/v FFP (Fresh Frozen Plasma—National Blood Transfusion Service). Culturing this biomass has been published in detail [4], as has its performance in an animal trial [30].

### Large volume cryopreservation protocol

A cryopreservation solution consisting of 24% DMSO (Sigma, Gillingham, Dorset) in 76% UW Solution (University of Wisconsin solution, Bridge to Life, Columbia, SC, USA) (v/v) was prepared and chilled to 4°C. 0.5g Icestart (Asymptote ltd., Cambridge, UK) was added as ice nucleant.

The 2.3 litre biomass was removed from the bioreactor, and added to this cryopreservation solution at a 1:1 ratio, mixed, and allowed to equilibrate for 15 minutes. After equilibration, the ELS had settled in the solution, and most of the supernatant was removed, leaving 2.3 litres of biomass (equilibrated to 12% DMSO and 38% UW solution) and 0.6 litres buffer supernatant. This gave a total volume of 2.9 litres. The ELS sank to the bottom of the cylinder under gravity, and so this extra medium buffer sat on top.

**Fig 1:**
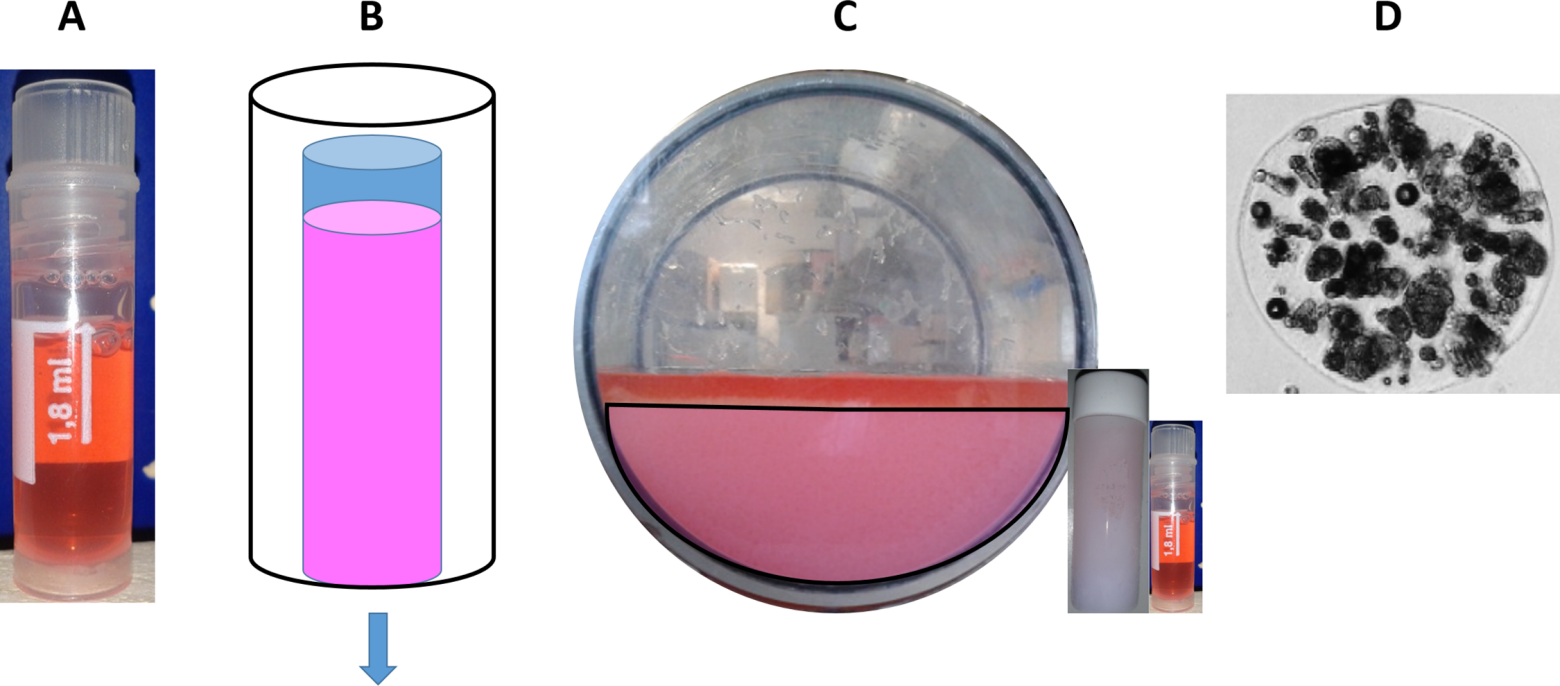
The three experimental designs tested. A–The cryovial set-up (CV). Samples were added to cryovials and cryopreserved as per the test conditions. B–The scale down process set-up (SDP). 6ml vials were filled with ELS. The vial was then insulated–indicated by the black outline–so that heat could escape only from the base of the vial–indicated by blue arrow. Samples in this set-up experienced the same conditions as a 2.9 litre volume on cooling, but were thawed rapidly. C–The large volume set-up (LV). 2.3 litres of biomass was added to the freezing chamber—indicated by the pink hemisphere–with 0.6 litre supernatant on top, indicated as red. A large air fraction existed above the biomass. The CV and SDP vials are shown to scale in C.

This volume (2.9 litres) was added to a 20cm long, 15cm diameter polycarbonate cylinder which was sealed with end caps. The cylinder was added to a Planer controlled rate freezer, and cooled at 0.3°C/min from 4 to -100°C. On reaching -100°C the chamber was removed from the freezer and stored for 5 weeks in the vapour phase of a liquid nitrogen reservoir, at approximately -170°C. The time taken between removal of the biomass from the bioreactor and the start of the cooling process was 20 minutes in all experimental conditions. The experimental set-up is shown in Fig 1.

### Scale down process protocol

To mimic the cryohistory experienced by the large volume in a scale down manner, an Asymptote EF600 with an added module was used. This module allowed conduction only through the base of 6-8ml vials. ELS were prepared and mixed 1:1 with the chilled cryopreservation solution outlined above (0.1g Icestart used per vial), before being cooled at 0.3°C/min from 4 to -100°C and plunged into liquid nitrogen storage.

The total volume per vial was 5 ml ELS and 1.25ml supernatant. This method has been published elsewhere in detail [6, 29], and can be seen in Fig 1.

### Cryovial cryopreservation protocol

A 2ml mix of 80% ELS equilibrated with the chilled cryopreservation solution and 20% buffer supernatant was added to 2ml cryovials and cooled at 0.3°C/min from 4 to -100°C and then plunged into liquid nitrogen storage [6]. 0.02g (1% w/v) Icestart was added to vials to induce ice formation. For the samples exploring the effects of warming rate that are presented in Fig 1, 0.1g of Icestart was added per vial to induce more PS (progressive solidification) per sample.

### Large volume thawing and re-culture protocol

**Fig 2:**
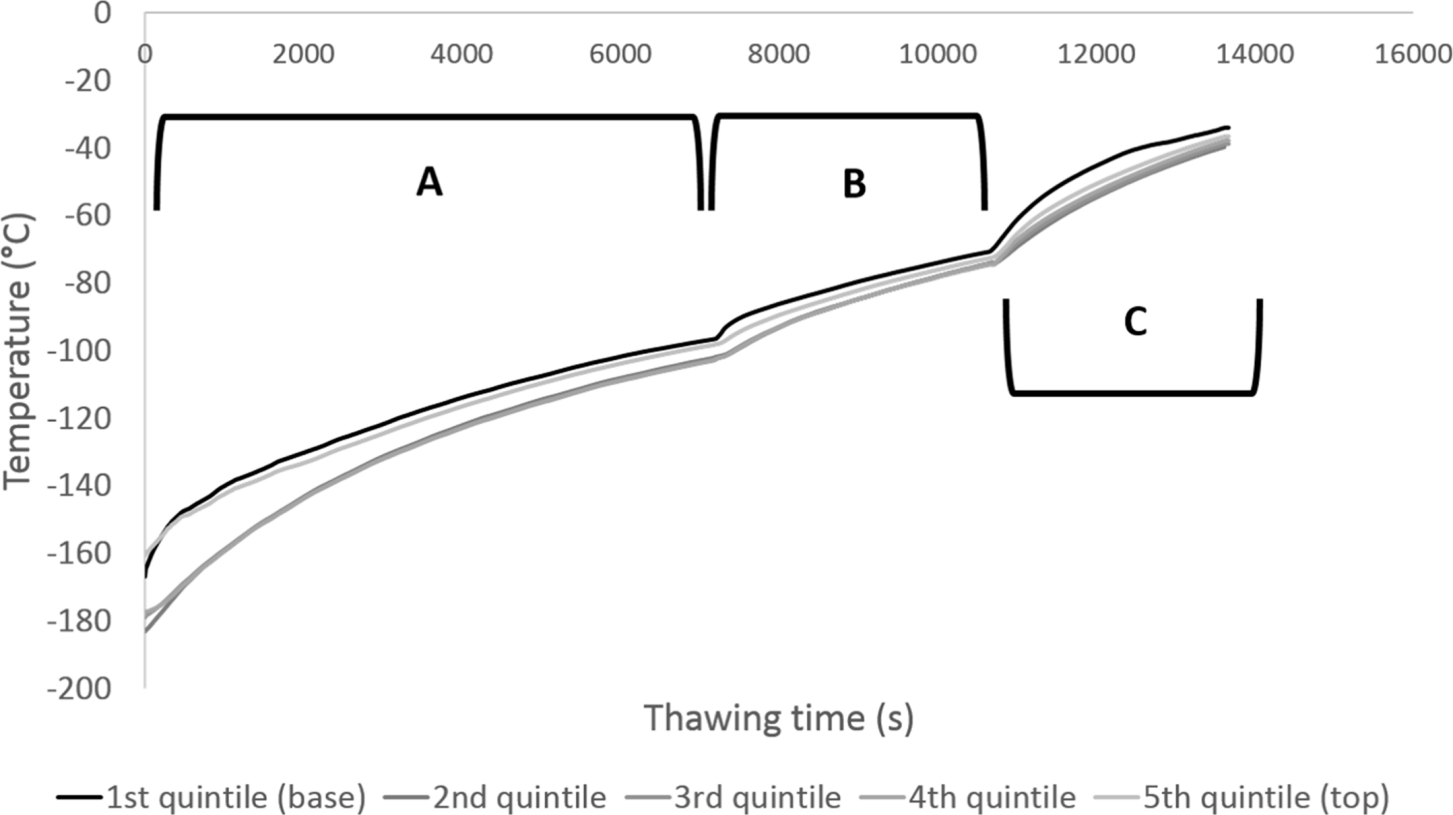
Warming profiles experienced during warming of the large volume cryopreservation cylindrical chamber. Thermocouples were placed at the bottom of the biomass (black) and the top of the biomass (lightest grey), as well as three others equidistant apart between the bottom and top following a straight line through the deepest part of the sample (dark to lighter grey). Section A demarks warming in the -80°C freezer, section B thawing in the -30°C freezer, and section C -10°C in the Planer controlled rate freezer. Relatively little intra-sample temperature variation was observed, a maximum of 22°C difference was observed at the start of the warming process, diminishing to 9°C intra-sample variability after the 4 h warming process. Average warming rate was 0.6°C/min.

The large volume cryopreservation cylinder was removed from the liquid nitrogen reservoir and placed into a -80°C freezer for 2 hours. It was then transferred to a -30°C freezer for 1 hour, and subsequently moved to the Planer controlled rate freezer set to a constant temperature of -10°C for 1 hour. This is highlighted in Fig 2.

The cryopreservation cylinder was then transferred to a large basin in a sterile hood where the end caps were removed and 20°C HBSS (Hank’s Buffered Saline Solution, Gibco, Paisley, UK) was added to the system. A total of 10 litres was added over 30 minutes, with the system stirred throughout to dilute out the DMSO and encourage melting. After all the ice had melted, the supernatant was removed and ELS added to the FBB. Approximately 2.1 litres were added to the bioreactor, with 200ml having been lost during the cryopreservation cycle. Post-thaw culture was performed for up to 120h with periodic sampling for functional analyses.

### Scale sown process and cryovial thawing and re-culture protocol

Vials were removed from liquid nitrogen and warmed rapidly in a water bath taking approximately 330 seconds to thaw. DMSO was diluted out in a stepwise manner at a 1:1 ratio every 2 minutes for 10 minutes. Samples were added to warmed culture medium and cultured in a Rotary Cell Culture System (RCCS, Synthecon) with a rotation rate of 10 rotations/min.

For scale down samples where the spatial dimensions were studied, the vials were warmed slightly by immersing into 37°C water for 25s and the frozen cores extracted from the samples. These were dissected and warmed rapidly separately. This method has been outlined in detail previously [6, 29].

For samples where the warming rate was studied samples were removed from liquid nitrogen and placed into a -80°C freezer, a -20°C freezer, and a 4°C fridge, at time points analogous to the large volume thawing protocol above with the fridge replacing the -10°C freezer step. At certain temperatures, defined using thermocouples in representative samples, sets of vials were removed and thawed rapidly in a water bath as above.

### Cell viability analysis

A viability assay was carried out using PI/FDA staining. 20μl PI (propidium iodine solution, 1mg/ml, Sigma) and 10μl FDA (fluorescein diacetate solution 1mg/ml, Sigma) were added to ELS and incubated at room temperature for 90 seconds. The ELS were washed once in PBS (Invitrogen) and then florescence at 617 nm (excitation) and 520 nm (emission) measured, with 1 s and 150 ms exposure for PI and FDA staining respectively. The total FDA intensity was compared under a phase-contrast microscope to the total PI plus FDA intensity using Nikon imaging software giving a cell membrane integrity and metabolic viability read-out [4, 6, 31].

### Cell counts

Cell viability only measures cells which physically survive the freezing process (though may not have survived functionally). To get a true reflection of outcome cell counts must be carried out to take account of cells destroyed and removed from the system during the cryopreservation process.

A known volume of ELS was removed from alginate using 16mM aqueous EDTA (Applichem, Darmstadt, Germany) solution before the ELS were disaggregated, cells lysed, and a nucleic count carried out using a nucleocounter system. As HepG2 cells are mononuclear, this equates to cell number.

### Glucose consumption and lactate production

Culture medium samples were taken throughout the culture process, and the glucose concentration measured with an Analox GM7 device using oxidase enzyme reactions (using Analox reagent GMRD-002A, Analox, London, UK). This was then related to glucose consumption per sample.

The Analox GM7 was also used for lactate measurements on medium samples employing an L-Lactate oxidase reaction with reagents GMRD 092A and GMRD 092B from Analox. This was related to total production per condition.

### Alpha-fetoprotein production

Alpha-fetoprotein production was quantified by sandwich ELISA (Enzyme-Linked Immunosorbent Assay) in medium samples. The primary and secondary human antibodies were acquired from AppliChem, products ab10071 and ab10072 respectively, with AppliChem A6935 being used for a standard. This was then related to total production per ml ELS in the system.

### Temperature profiles

To determine temperature profiles during warming of the large volume biomass, a thermal mimic was set-up using 10% (v/v) glycerol in water, which we have established has the same thermal properties as ELS in the freezing mix [6].

An incision was made in the top of the chamber, into which k-type thermocouples (Pico-technology, St. Neots, UK) were added at set depths in the solution. The cylinder was then cooled in a large liquid nitrogen reservoir, before undergoing the warming protocol detailed above for the 2.3 litre biomass. The thermocouples were attached to a picologger unit and recorded using picotechnology software.

### Statistics

To determine significance, an appropriate Student’s t-test was performed. Significance was determined at P<0.001 unless otherwise stated. Samples for cell functional analysis contained five replicates unless indicated differently.

## Results

### Warming rate significance

**Fig 3:**
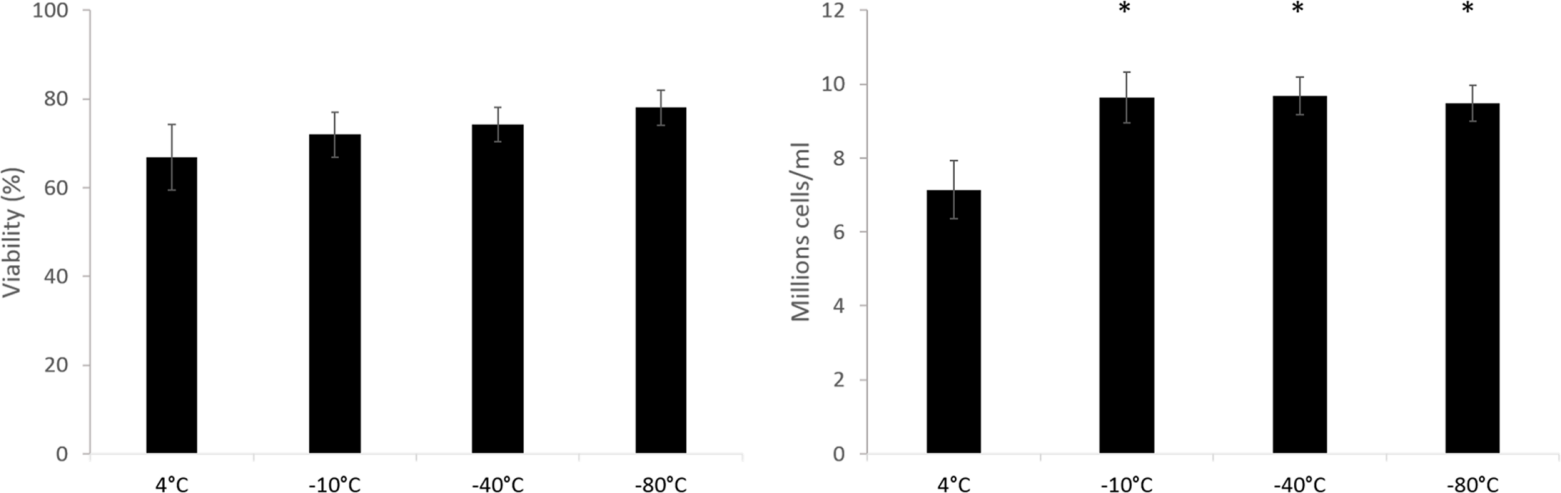
Viability (left) and viable cell number (right) of samples being warmed at 1°C a minute from -196°C, to the temperatures indicated, before being warmed rapidly to thaw in a water bath (in 2ml cryovials). Data 24 h post-thaw. The rightmost sample (4°C), was warmed slowly until thaw. No significant difference was seen in viabilities. All samples warmed rapidly through the phase transition had significantly increased viable cell number over the samples warmed slowly (4°C sample), viable cell number was 16.3 ± 1.7 million cells/ml immediately prior to cryopreservation. N = 5 ± SD, significance defined as * = P<0.001, unpaired Student’s t-test.

It was determined using cells cryopreserved in vials that the warming rate need only be controlled in the final phase of the thaw (Fig 3). Samples that were warmed slowly from liquid nitrogen to -80°C, and then rapidly until thawed exhibited no significance difference in viable cell number 24 h post thaw compared with those warmed slowly from LN_2_ to -10°C before being thawed rapidly. With regards to ice melting, samples that were warmed slowly from LN_2_ and slowly through the phase transition had significantly lower viable cell number 24 h post-thaw.

### Buffer media significance

**Fig 4:**
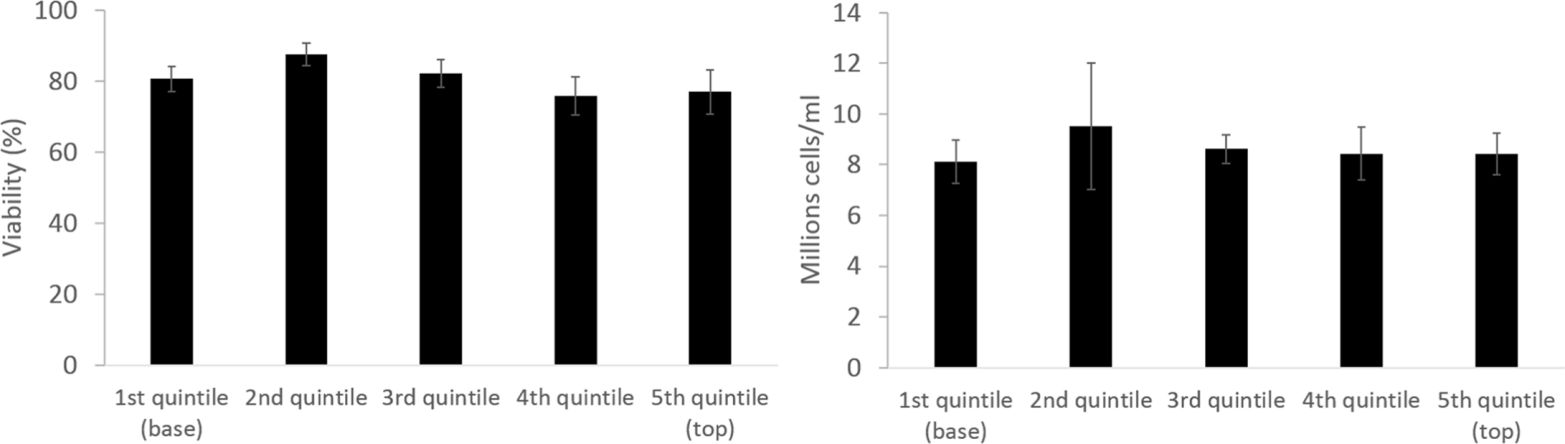
Viability (left) and viable cell number (right) of samples cryopreserved with 20% of the volume excess medium (in 2ml cryovials). 24 h post-thaw no significant difference was observed in either viability or viable cell number between any sampling location. viable cell number was 18.2 ± 1.6 million cells/ml immediately prior to cryopreservation N = 5 ± SD.

Fig 4 demonstrates that there was no discernible spatial significance 24 h post-thaw in samples cryopreserved with 20% of the total volume acting as a buffer supernatant. While this extra volume would increase the total melting time of large volume samples, this was more than mitigated by improvements the buffer region gave.

### Large volume warming profile

The warming data in Fig 2 shows that the large volume sample experienced relatively little intra-sample variation during the warming phase, indicating that the main barrier to heat transfer was the 3mm polycarbonate wall of the chamber, not the conduction through the solidified mass. The average temperature in storage was -173 ± 9°C, which rose to -37 ± 2°C after the 4 hour warming profile—this approximately halved the energy required to thaw the device.

While the -80°C and -30°C freezers contained only static air, the Planer freezer at -10°C has a fan to circulate air, hence the relatively rapid warming in this phase.

### Viability and viable cell number

Prior to cryopreservation, all samples had a viability of 99.6 ± 0.1% and a viable cell number of 30.9 ± 1.7 million cells/ml.

**Fig 5:**
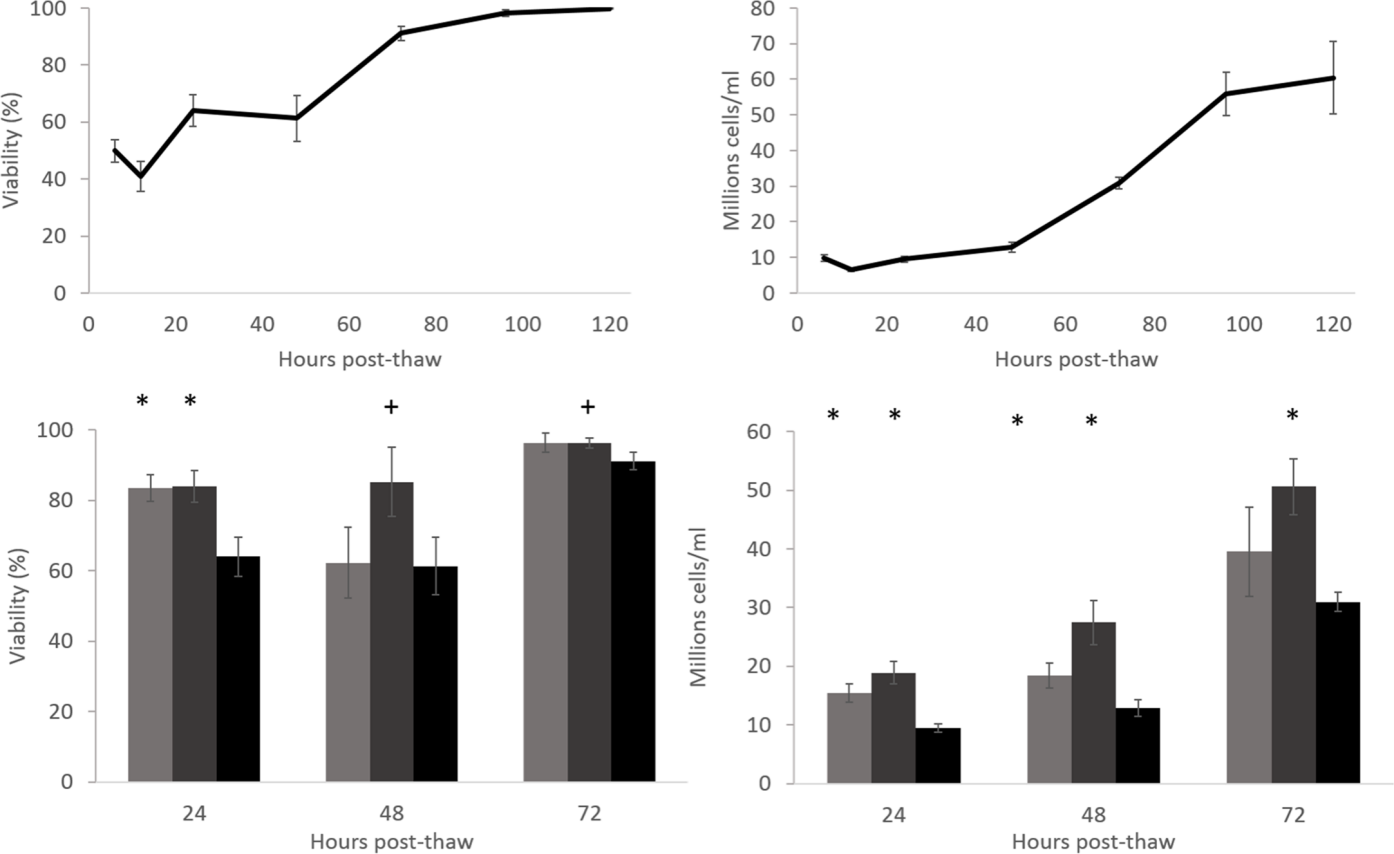
Top; viability (left) and viable cell number (right) post-thaw of the large volume cryopreservation. Base: Viability (left) and viable cell number (right) of cryopreserved cryovials (CV), scale-down process vials (SDP), and the large volume (LV) (light grey, dark grey, and black respectively). The viability was significantly higher in the scale-down process vials compared to the large volume cryopreservation at all measured timepoints, and was significantly higher in the cryovials over the large volume at 24 h post-thaw. Both the cryovial and scale down process vials samples had significantly higher viable cell number over the biomass at 24 and 48 h post-thaw, the scale-down process vials significantly better also at 72 h post-thaw. N = 5 ± SD, significance over large volume cryopreservation defined as * = P<0.001, + = P<0.005 unpaired Student’s t-test.

24 hr post thaw the viable cell number fell to one third of the pre-freeze values (Fig 5) the LV samples recovered to their pre-thaw viable cell number 72 h post-thaw with 31.0 ± 1.6 million cells/ml. Viability by 72 h post thaw was 91.1 ± 2.5%, recovering to 99.8 ± 0.1 120 h post-thaw.

Samples cryopreserved using either the CV (Cryovial) or SDP (Scale Down Process) methods recovered more rapidly, CV samples displayed a viability and viable cell number of 96.3 ± 2.7% and 39.5 ± 7.6 million cells/ml 72 h post-thaw respectively. SDP samples recovered most-rapidly and exhibited 96.2 ± 1.5% and 50.6 ± 4.8 million cells/ml viability and viable cell number respectively by 72 h post-thaw, comfortably exceeding the pre-cryopreservation values.

It was noted that while 2.1 litres of ELS was added to the bioreactor, 2.3 litres was recovered at the end of the experiment post-thawing, indicative of the diameter of the ELS swelling by 4–5% during the culture period.

### Glucose consumption

**Fig 6:**
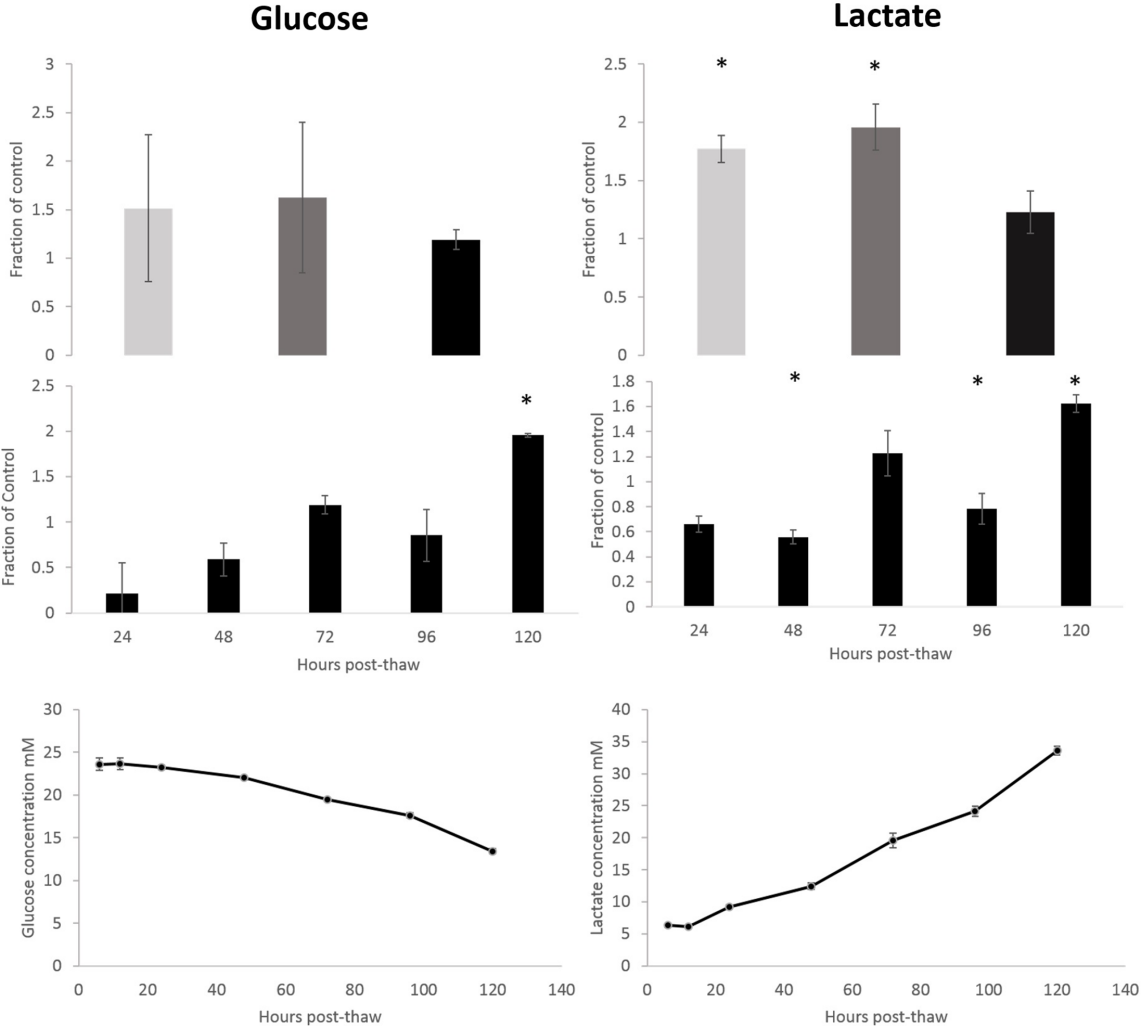
Glucose consumption (left) and lactate production (right) following cryopreservation. Top–glucose consumption and lactate production per ml ELS as a fraction of unfrozen control 72 h post-thaw, comparing the cryovial cryopreservation (light grey), scale-down process cryopreservation (dark grey), and large volume cryopreservation (black). No significant difference was observed in glucose consumption. Lactate production was significantly higher in both the cryovial and scale-down process samples over the large volume cryopreservation. Centre–production at various timepoints in the large volume cryopreservation per ml ELS over an unfrozen control. Base–Total glucose and lactate measured in the bioreactor at set timepoints post large volume thaw. N = 5 ± SD, significance over large volume cryopreservation (top) or unfrozen control (centre) defined as * = P<0.001, unpaired Student’s t-test.

24 hours after thawing the glucose consumption per ml of ELS was significantly reduced in the large volume (to 22% per ml ELS) compared with the unfrozen controls. 72 h post thaw, samples cryopreserved in all conditions were consuming at least as much glucose per ml ELS as before cryopreservation (Fig 6). No significant difference was observed between the experimental conditions. In the LV condition, glucose consumption was significantly (95%, p<0.001) higher 120 h post-thaw than pre-cryopreservation per ml ELS. A general decrease in glucose in the culture medium was also observed throughout the re-culture period, falling to a minimum of 13.4mM from 23.6mM from 6 to 120 h post-thaw. 120 h thaw large volume samples were consuming 3.5 ± 0.1 mM per 24 h compared with 3.5 ± 0.4 mM pre-freeze.

### Lactate production

24 h after thawing the lactate production per ml of ELS in the LV set-up was significantly reduced compared with the unfrozen controls (to 65% per ml ELS). 72 h post-thaw, samples cryopreserved in all conditions were producing at least as much lactate per ml ELS as before cryopreservation (Fig 6). Significantly higher quantities were produced per ml ELS in samples cryopreserved in the CV and SCP experiments over either LV samples or pre-cryopreservation values. In the LV condition, the lactate production was significantly lower at all timepoints per ml ELS compared with the pre-cryopreservation values, except at the 72 h time point where no significant difference was observed, and at 120 h post-thaw where lactate production was significantly higher. Lactate concentration in the large volume bioreactor was observed to increase throughout the culture period, from 6.1mM to 33.1mM from 12 to 120 h of re-culture, analogous to the decrease in glucose concentration. 120 h thaw large volume samples were producing 7.9 ± 0.7 mM per 24 h compared with 9.4 ± 0.3 mM pre-freeze.

### Alpha-fetoprotein production

**Fig 7:**
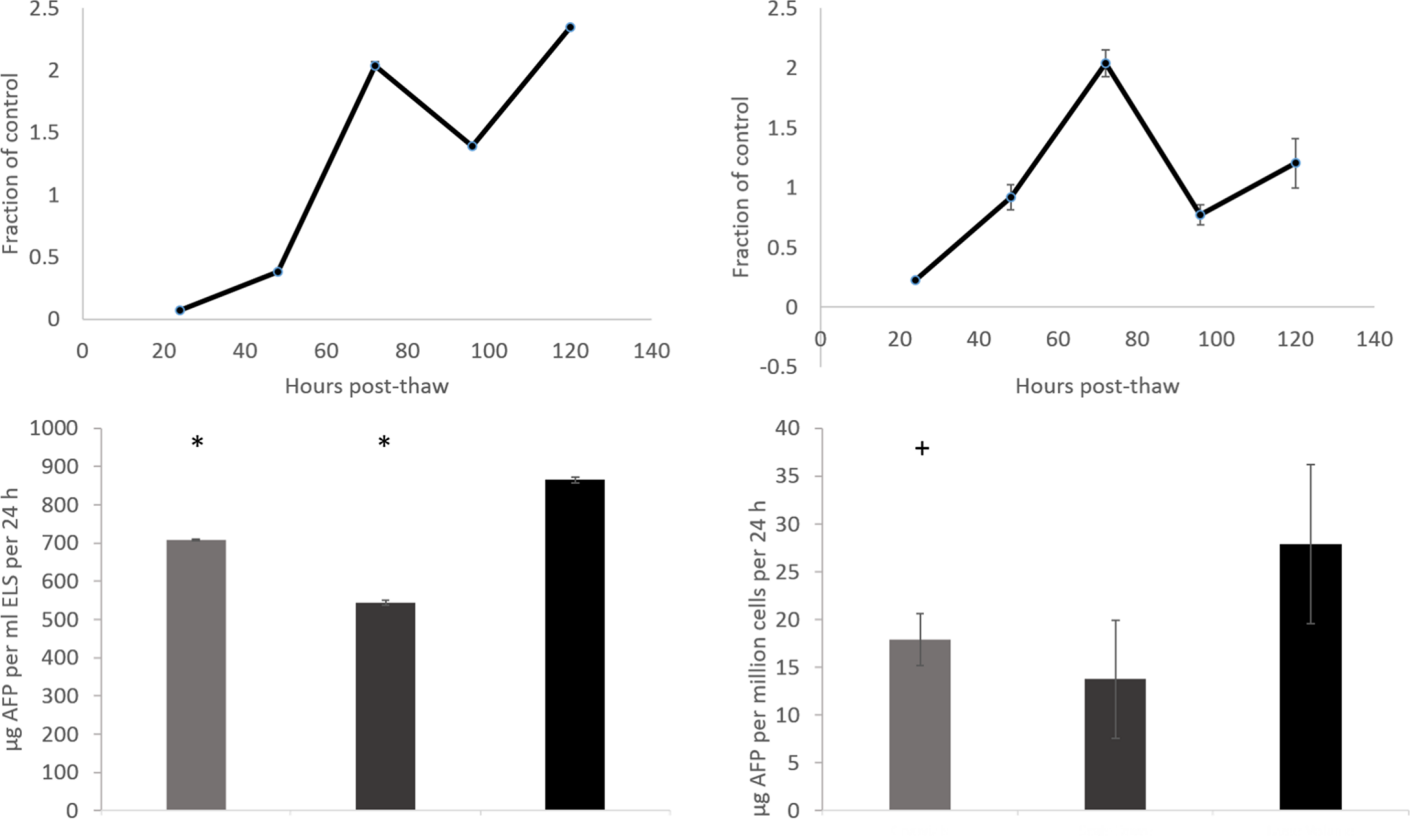
AFP production of large volume cryopreserved samples, and comparisons with cryovials and scale down process samples. Top: production at timepoints post-thaw as a fraction of unfrozen control, either per ml ELS (left), or per million viable cells (right). Base: comparison between cryovials (light grey), scale down process samples (dark grey), and large volume samples (black) at 72 h post-thaw. Per ml ELS, both conditions produce significantly less AFP than the large volume sample, and per million cells the cryovial production is significantly lower compared with the large volume cryopreservation samples. N = 5 ± SD, significance compared to large volume cryopreservation (top) defined as * = P<0.001, + = P<0.005, unpaired Student’s t-test.

Alpha-fetoprotein (AFP) production used here as an indicator of wider protein production (that cannot be accurately determined in this system due to the inclusion of human blood fraction in the culture medium), had recovered to pre-cryopreservation levels in the LV set-up 48 h post-thaw (Fig 7). This holds when considering either production per ml ELS or per million viable cells.

Per ml ELS, samples in the LV set-up produced significantly (P<0.001) more protein per ml ELS than CV or SDP samples 72 h post-thaw. LV protein production was also significantly upregulated per million viable cells over CV samples (P<0.005) 72 h post thaw. 120 h thaw large volume samples were producing 16.5 ± 0.2 μg per million cells per 24 h compared with 13.7 ± 0.3 μg per million cells per 24 h pre-freeze.

## Discussion

### Warming rates significance

This study examined three separate conditions. First, those of samples cryopreserved in cryovials that experience network ice solidification on cooling and then warmed rapidly (CV). This represents what happens during small volume cryopreservation. Second, samples cooled using a scale down process that experienced progressive ice solidification on cooling and were warmed rapidly (SDP). This represents samples experiencing similar conditions to large volumes on cooling but similar to small volumes on warming. Finally, that of a cryopreserved 2.9 litre volume containing 2.3 litres of biomass that experienced progressive ice solidification on cooling and was thawed relatively slowly (LV). This represents large volume cooling and warming.

Previous studies and protocols using hepatocytes employ rapid warming for optimal cryopreservation strategies [8–11, 32]. Most of these studies have only focused on small volumes and have not explored slower warming rates. Avoidance of recrystallization is often cited as a reason that rapid thaw is required for cell types. As this is not a problem during slow progressive solidification where samples already have large ice crystals, and slow warming is typically acceptable after slow cooling rates [33].

The study (Fig 3) indicated that during the warming phase, the rate of ice melting (time between visible macroscopic melting and the last ice crystal disappearing) is more important than overall warming rate to post thaw recovery. Rapid warming is only necessary during the melting phase of ice; ice does not melt uniformly on the microscale, but melts in some places and forms in others depending on localised heat and mass transfer, and macroscopic thawing occurs where more areas of ice are receding than growing.

**Fig 8:**
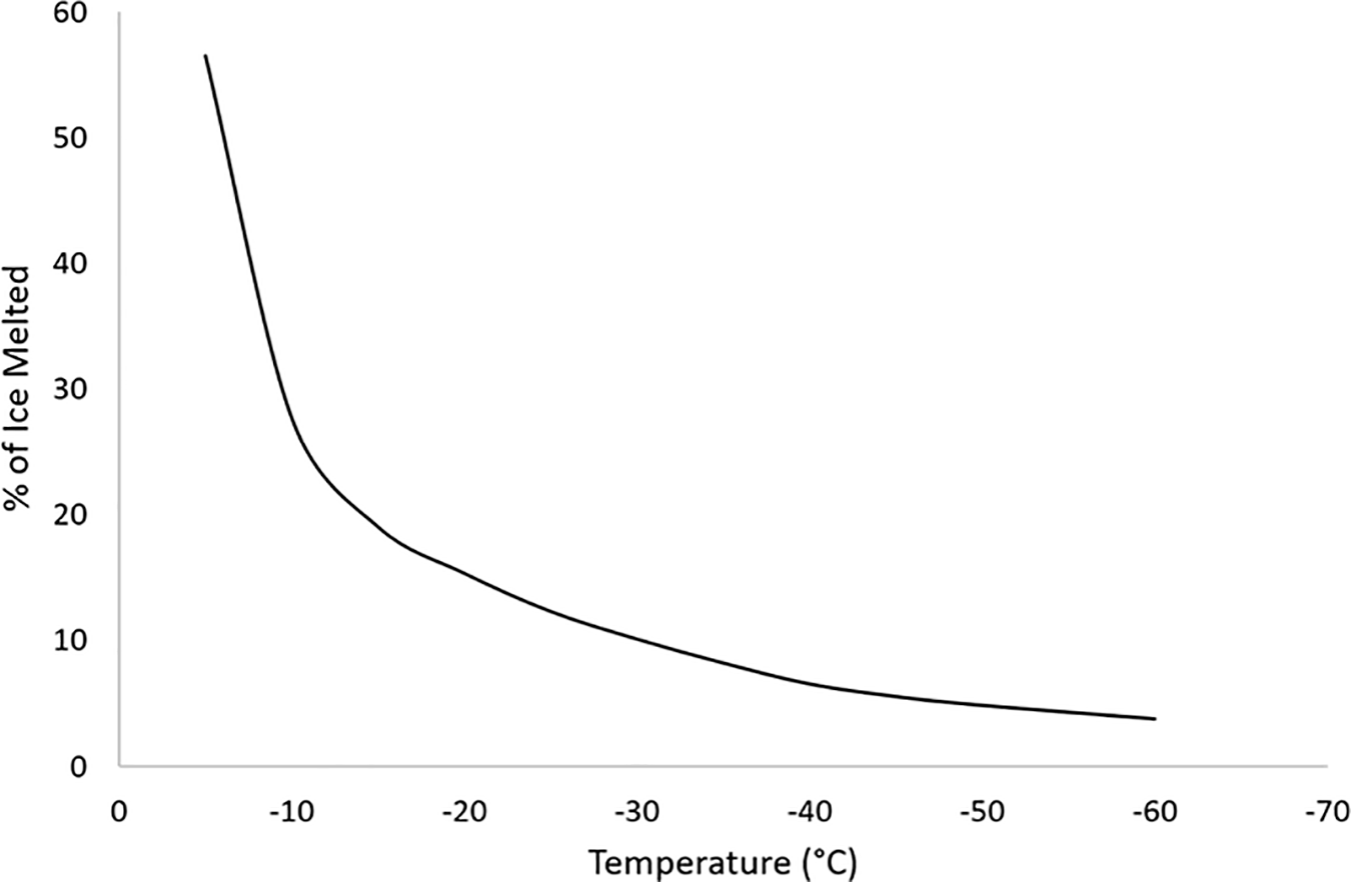
The proportion of extra-cellular ice melted at different temperatures during the warming phase. Below the glass transition temperature (-120°C) all extra-cellular water is either ice or is vitrified in solute concentrated channels between the ice crystals. On warming the ice crystals melt due to the freezing point suppression of the high solute concentrations, a process which accelerates at high sub-zero temperatures until complete thaw occurs at -4.5°C. Data for a 12% v/v aqueous DMSO solution and adapted from Rasmussen and MacKenzie [48].

This was an encouraging finding as it allowed the sample to be warmed slowly without damage to the cells before transfer through the equilibrium melting point. Thawing the 2.9 litre volume containing the 2.3 litres of biomass rapidly (<30 mins) from LN_2_ to the liquid state would have been difficult to achieve practically due to the poor heat conduction through the chamber walls (3mm polycarbonate) and the distance from the centre of the sample to the chamber walls which substantially slows heat transfer rates. It should be noted that small amounts of liquid will be present in channels between the ice from the glass transition temperature (T_g_) until the equilibrium melting point through the melting of the freeze-concentrated channels. T_g_ in an aqueous DMSO solution is -120°C [34]. This melting will be non-linear, with the bulk of the melting happening close to the equilibrium melting point [29, 35]. Fig 8 shows the approximate portion of ice which has melted at any given temperature during the warming cycle.

Our hypothesis is that during melting, cells in the melted phase experience additional DMSO toxicity, and therefore this is the critical area to control during the thawing of large scale structures.

While the equilibrium melting point of our solution is -4.5°C [5], as discussed above the freeze-concentrated channels will start to thaw prior to that point (Fig 8) [36]. Much of the sample will leave the solid phase before this temperature is reached although this may not be visually apparent on the macroscale [37, 38]. The thaw temperature was therefore chosen as below -30°C, to mitigate any damage that may occur cellularly to the sample of extended time (hours) in this state.

There has been very little focus on the impact of warming rates of samples cryopreserved using slow rates of freezing (<1°C/min), with ice seeding to induce progressive solidification which is relevant here [8, 39, 40]. In general samples cooled rapidly must be thawed rapidly, while those cooled slowly can be thawed either slowly or rapidly for optimal outcome [33].

It is important to note that much of the cryobiology literature exploring warming rates only looks at average warming rates over the whole process. For example, fast warming is induced by placing samples into a 37°C water bath, and slow warming rates by placing the samples in chilled air. Warming rates will not be linear in these situations, rather samples will warm rapidly to start with, with the temperature asymptotically reaching the environment temperatures (with a discontinuity through the phase-transition).

If warming rate were defined as the average of the warming rates between each 1°C increment of warming, then the average warming rate would be found to be much higher than the stated values since warming through most temperatures, say -200 to -50°C, would be much more rapid than -50°C to 0°C where slower warming occurs. On the other hand, if the process were taken to be time-dependant, the average warming rate would be much lower than stated, as most of the time is nearer the environmental temperature, where the warming rate is relatively slow.

The closer to linear the warming rate is, the smaller the difference in these values becomes. Stating an average warming rate for an asymptotic warming profile assumes that time and temperature are equally important during the warming phase, although there is little published data to support this.

### Buffer media significance

As ice develops through a volume, solutes will concentrate in the last areas to solidify. Since cells are sensitive to cryoprotectant concentration in a system, this leads to a deviation from optimal concentrations and is harmful to biomass solidifying later within a large sample [29]. In addition, the later that cells solidify in a larger volume, the greater their rate of cooling post-solidification. This is a consequence of heat transferring through the solid state much more easily than the liquid state, and later solidification requires more ‘catching up’ to the external controlled rate freezer temperature [6, 28, 35]. This is compounded by the release of latent heat on solidification deep within the sample. Cells are sensitive to cooling rates, and so this will also be detrimental to a greater degree in a large sample.

To overcome this cryoconcentration, 20% total volume of the sample was ‘buffer’ liquid (2.3 litres biomass with 0.6 litres excess buffer supernatant) to allow extra bulk for solutes to increase into away from the biomass. The ELS are weighted with glass beads so that this medium is found above the biomass, further from the chamber walls on average, and resulting in it solidifying last. While this increased the total energy required to thaw the system [8], it was more than mitigated by reducing cryoprotectant toxicity and non-optimal cooling rates.

This 20% buffer supernatant was found to remove damage observed when only 9% total volume (ELS+ 10%) is medium when tested in the SDP [6].

### Recovery timeframe

Viability recovered more slowly than would be seen in cell suspensions or monolayers due to cells being encapsulated within the alginate and so dead cells were not removed through wash-outs. Success post-thaw was defined as a viability >90%. The low nadir of recovery comes 6-24h post-thaw, an expression of delayed onset cell death.

The 2.3 litre biomass had recovered lactate production, glucose consumption, viable cell number, and AFP production to pre-cryopreservation values within 72 h of thaw. Viable cell number reached a nadir 12 h post-thaw, which is typical with apoptosis induced delayed onset cell death in cryobiology [41, 42].

Comparing with CV and SDP samples, the most successful conditions during the cryopreservation cycle are those that experience progressive ice solidification on cooling, but are thawed rapidly. These are likely better than those cooled in cryovials which will experience a much greater degree of network solidification and so are susceptible to re-crystallization on thaw and also experience undercooling and sharp temperature discontinuities on ice nucleation [6].

This study established quite clearly the need for more rapid melting techniques. Slow warming for most of the process helps to reduce this time, but significant energy must be provided for the final thaw step, and the slow warm must stop prior to the equilibrium melting point due to the reduction in ice fraction prior to this temperature. The melting time was reduced to 30 minutes in this study for the large volume, and further reductions in this time would improve biomass recovery.

The LV sample showed robust protein production recovery post-thaw compared with CV and SDP samples, indicating that the cellular structures involved in AFP production were less affected by the warming profile and may in fact prefer slower warming rates. If the melting time could be reduced to around 5 minutes, the SDP samples showed that post-thaw viability, viable cell number, and lactate production, could be significantly improved, indicating that these functions were particularly sensitive to warming rates. Methods such as radiofrequency warming or dielectric warming are the most promising avenues to explore to further improve post-thaw recovery [43–46].

The field of cryopreservation has largely focused on smaller volumes, which typically undergo different cryostresses relative to larger volumes. Indeed many published studies tend to use the term ‘large volume’ with samples larger than around 5ml. Large volume cryopreservation, on the order of litres, requires a step change in cryobiology thinking and is required for preservation of organs and tissue engineered constructs, for which Just-In-Time manufacture is not possible [7, 8, 19].

It is important to highlight the form of cell recovery. For the large volume samples, viable cell numbers fall to around 10 million viable cells/ml ELS (from an initial cell density of 30.9 ± 1.7 million cells/ml), and the cell viability (biological viability of cells which physically survive freezing), is 60% compared with 99.6% before cryopreservation. Due to the speed of the recovery, it is likely that cells identified as non-viable by dye binding are in fact transiently membrane permeable, as they recover quickly (non-viable defined through cell membrane permeability to propidium iodide), such that they become membrane impermeable again, with improved viability confirmed through functional and dye binding tests. It is also clear that cell-proliferation makes a large contribution to the recovery timeframe, with new cells replacing those damaged beyond repair during the cryopreservation process. Optimising the cryopreservation process also optimised this cell replacement process.

It is also important to note the differences in initial recovery as indicative of different forms of damage to cell pathways. Glucose consumption and lactate production is reduced and in the large volume sample compared with the other conditions. The lower initial consumption may indicate gluconeogenesis by hepatic cells in some ELS, before proliferation increases and glucose consumption likewise rises; the higher consumption in CV and SDP samples is consistent with the observed higher cell recovery at earlier timepoints, and previously observed results [4]. On the contrary AFP production is relatively less impacted by the cryopreservation process with the large volume indicating that while fewer cells survive the cryopreservation process in the large volume, those which do tend to be healthier. Note that AFP was used as a marker for protein production as albumin and other human proteins are found in the culture medium plasma, making differentiation of BAL proteins from medium proteins difficult, we have previously carried out tests comparing protein production [6]. Different functions being impacted differently when cryopreservation protocols are varied has been observed in the cryopreservation of ELS previously [6]. Culturing sufficient biomass from encapsulation takes 12 days, plus additional days to produce sufficient cells for encapsulation, all together requiring ∼26 days; the culture-conditioned product cannot be stored for any more than a few days without cryopreservation with these optimised parameters [47]. Carrying out this cryopreservation process will reduce the timeframe for starting BAL treatment of ALF from 26 to 3 days, making the treatment delivery logistically capable of meeting clinical needs.

## Conclusions

To summarise, we have demonstrated that adding extra culture medium and having a slow warming phase followed by a rapid thaw allows the cryopreservation and recovery within 72 h of a 2.3 litre bioartificial liver biomass. This is the largest volume that has been cryopreserved and thawed in a single cassette reported in the literature to date and would be a precondition for the bioartificial liver device to be available on demand. Further reductions in thawing time could reduce the delivery timeframe to 48 h.

## Supporting information

S1 File (S1_Raw_Data.xlsx). The raw data for Figs 2–7.
(XLSX)

## Abbreviations

ELS: Encapsulated Liver Spheroids
PS: Progressive Solidification
NS: Network Solidification
BAL: Bioartificial Liver Device
UW: University of Wisconsin Solution (Viaspan)
